# Effect of physical mobility, decision making and economic empowerment on gender-based violence among married youth in India-SAWERA project

**DOI:** 10.1186/s12889-023-15421-4

**Published:** 2023-03-23

**Authors:** Devika Mehra, Shobhit Srivastava, Murari Chandra, Namita Srivastava, Mari Laaksonen, Heidi Elina Saarinen, Sunil Mehra

**Affiliations:** 1 MAMTA Health Institute for Mother and Child, New Delhi, India; 2 Division of Social Medicine and Global Health, Department of Clinical Sciences, Lund University, Malmö, Sweden; 3 Physicians for Social Responsibility, Helsinki, Finland

**Keywords:** Sexual violence, Physical violence, Women’s empowerment, Youth

## Abstract

**Background:**

Preventing and responding to gender-based violence (GBV) is both a human rights imperative and a multifaceted economic issue. GBV can also act as a barrier to economic empowerment. The aim of the study was to examine the association between women’s empowerment (physical mobility, decision making and economic resources) and GBV among married youth in India.

**Methods:**

Community based cross-sectional study was conducted among married youth in the age group of 15–24 years, in two selected districts of Uttar Pradesh and Rajasthan, India. The data was collected from 578 youth. Pre-validated scales were used to assess women’s empowerment indicators (physical mobility, decision making and economic resources). The outcomes assessed were scales on physical and sexual violence. Multivariate regression models examined associations between women’s empowerment, spousal characteristics, socio-economic status and demographics.

**Results:**

The overall results of the study found that restricted physical mobility had a negative association with sexual violence [AOR: 0.49; CI 0.26–0.92]. Women with no decision-making power had higher odds of physical violence [AOR: 2.12; CI 0.01–4.43] and sexual violence [AOR: 1.96; CI 1.02–3.77]. Having no economic resources had a negative association with sexual violence [AOR: 0.19; CI 0.09–0.39]. Women going through spousal controlling behavior had a higher likelihood of physical [AOR: 3.79; CI 1.75–8.19] and sexual violence [AOR: 4.03; CI 2.09–7.79]. It was also found that married women from rural areas and other ethnic backgrounds had higher odds of physical violence.

**Conclusion:**

There is a crucial need to work towards women’s empowerment, with progressive gender roles such as greater decision-making, physical mobility and economic resources to reduce GBV. An established method that has worked in various contexts is adopting gender transformative approaches that involve men.

**Supplementary Information:**

The online version contains supplementary material available at 10.1186/s12889-023-15421-4.

## Introduction

Violence against women (VAW) and girls is a global phenomenon that historically has been hidden, ignored, and silently accepted [1]. Globally, one in three women experience violence in her lifetime. Preventing such violence has been a priority of World Health Organization (WHO) since 2013 [2], and is a target for the fifth Sustainable Development Goal (SDG) [3]. Women continue to suffer physical, emotional, sexual, and economic violence [4].

Global research, prevention and intervention efforts continue to focus on physical and sexual intimate partner violence (IPV), negative health outcomes, including Human immunodeficiency virus (HIV), sexually transmitted infections (STI), unintended pregnancy, addictions and mental health issues remain [5, 6]. Evidence also suggested interventions like transformation of gender norms through behavioral change and communication-focused programs can avert GBV and VAW [7, 8].

IPV commonly used interchangeably with Gender-based violence (GBV), is the most common form of violence globally, particularly in India [2]. According to the Indian National Family Health Survey (NFHS -5) prevalence of spousal violence/IPV is 29.3% [9] which is substantially higher compared to other developing countries. Evidence from recently released national data (National Crime Records Bureau, 2020) suggests that approximately 30% of all documented crimes against women in India were attributable to ‘cruelty by the husband or his relatives’ [10].

India has the largest number of child marriages than in any other nation [11], with 27.9% of the girls aged 20–24 years married by the age of 18 [9]. Despite, the progress made in increasing literacy in women and substantial overall development, the reduction in the number of child marriages over the years has been inadequate, amid the deep rooted gender inequalities and biased gender norms in the country [12]. Traditional gender roles and beliefs that restrict women’s mobility, marital choice, as well as the controlling behavior of husbands, relegate value of women to domestic contribution and motherhood, often limiting women’s decision making power, men’s share in household chores, and justifying VAW in marriages [13].

Gender power dynamics is common to all intimate relationships. However, the dynamics is more challenging when there are spousal age differences [14]. Therefore, child marriage or controlling behavior by the older spouse especially in rural areas in India could lead to girls’ school dropout, mobility restrictions and lack of economic empowerment. Studies show that controlling behavior by men is significantly associated with higher likelihood of physical violence and sexual violence, given that controlling behavior reflects a power motive [15, 16]. Further, controlling behavior in intimate relationships can take various forms that can affect the well-being of the other partner. Most often it is the woman.

There is prior evidence showing that equally dependent partners who adopt egalitarian decision-making and have an equal division of power within the family most often report low levels of conflict, control, and violence [17]. Hence, moving in this direction, we chose to assess more progressive gender roles and beliefs than traditional gender roles among young married men and women in two states of India (Rajasthan and Uttar Pradesh), which has high prevalence of GBV. We feel looking into progressive gender roles, physical mobility (freedom of movement), decision making power, economic resources, safety within marriages (i.e. physical or sexual violence) is critical as looking into these aspects would help in highlighting the need for attitudinal and social norm changes to improve agency of women within marriage [18]. The current study aims to establish the association between empowerment indicators (physical mobility, decision making and economic resources) with GBV (physical and sexual) with respect to gender differentials (by which we mean that gender disaggregated data of men and women for all factors against GBV will be provided). We investigated with a gender lens as we anticipated that the pattern of violence among men and women would be quite different in a married context.

### Research questions:

1. Are women and men’s empowerment indicators (physical mobility, decision making and economic resources) associated with GBV?
2. What is the association between individual, socio-economic and spousal controlling factors that can lead to GBV among married youth?

### Conceptual framework

**Fig. 1:**
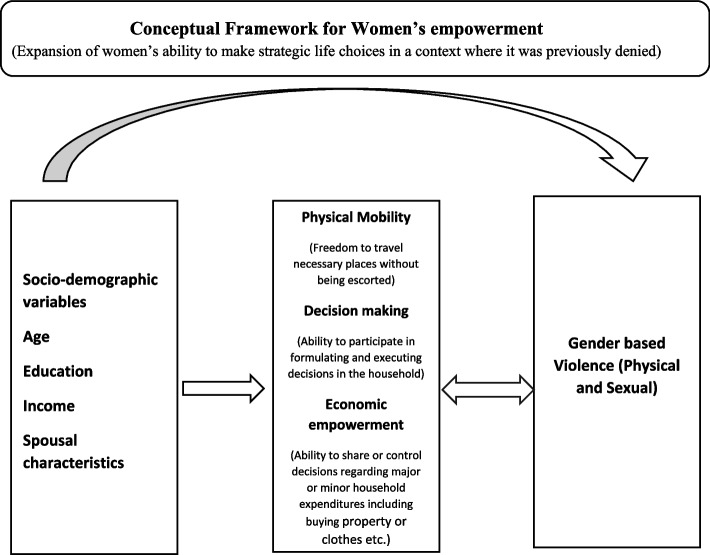
Conceptual framework for Women’s Empowerment

The conceptual model in this study has been adapted from the Women’s Economic Empowerment (WEE) Framework which is mentioned below in Fig. 1 [19]. It has been previously used in similar contexts for outcomes such as contraceptive use [19], violence [13] etc. The conceptual model is based on the premise that socio-demographic factors such as age, educational status of the respondent, income, spousal characteristics (age differentials, alcohol consumption) are associated with GBV (physical and sexual). Additionally, in a married context controlling factors by the spouse that restrict a women’s or man’s physical mobility, decision making and restricting access to economic resources is associated with GBV.

### Project description

Project SAWERA aims to address GBV by improving knowledge of youth (15–24 years) on sexual and reproductive health and rights as well as generating awareness on gender equitable norms and practices.. The project has adopted a Gender Transformative Approach (GTA) which is used to empower and capacitate the young peer educators while working on biased social norms that leads to Gender based violence including IPV. The project intends to fill a research gap by creating evidence on the prevalence and experiences of GBV among youth (girls and boys) and linking them to the GBV related health services, victim support and legal services. The results represented in the current study are derived from the baseline findings of the SAWERA project.

## Methods

### Study design and setting

The study adopted a cross-sectional design among married youth aged 15–24 years from March 2021 to Mid-April 2021. The data of this study is derived from the baseline study of the SAWERA project. The project interventions are based in two selected districts, Baharaich in the state of Uttar Pradesh (rural) and Jaipur in the state of Rajasthan (urban) in India, from 2021–2024. The population of Baharaich and Jaipur is approximately 3.4 million and 6.6 million respectively, according to census 2011. The coordinates for Baharaich and Jaipur are 27.575°N 81.594°E and 26.9°N 75.8°E respectively.

### Measures

#### Outcome variables

*Physical violence* was assessed using the following questions: Has your husband/partner ever A) Slapped you or thrown something at you that could hurt you? B) Pushed you, shoved you or pulled your hair? C) Hit you with his/her fist or with something else that could hurt you? D) Kicked you, dragged you or beat you up? E) Choked or burnt you on purpose? F) Threatened to use or actually used a gun, knife or other weapon against you? The responses of the above question were coded as 0 “no” and 1 “yes”. If the respondent responded 1 “yes” on any of the question, then he/she was coded into the category1 “experienced physical violence”; otherwise, 0 “not experienced physical violence”.

*Sexual violence* was assessed using the following questions: Did your husband/ partner ever A) physically forced you to have sexual intercourse when you did not want to? B) Have sexual intercourse when you did not want to because you were afraid of what your partner or any other partner might do? C) Have sex without a condom (but you wanted to use a condom)? D) Force you to do something sexual that you found degrading or humiliating? The responses of the above questions were coded as 0 for “no” and 1 for “yes”. If the respondent responded 1 “yes” in any of the question, then he/she was coded in the category 1 “experienced sexual violence”; otherwise, 0 “not experienced sexual violence”.

### Explanatory variables

Construction of women’s empowerment indices comprised of physical mobility, decision making power and economic resources.

Physical mobility (Cronbach alpha: 0.90): The variable was assessed using the following questions: Have you ever been there alone? Have you ever been to the hospital/clinic/health centre/health post/doctor alone? Have you ever gone to a place for community entertainment, such as a cinema/video/film house/sport games etc. alone? Have you ever gone to a religious place (temple/church/mosque) alone? Have you ever gone outside the neighborhood/village alone? The responses of the above question were coded as 0 “with someone” and 1 “alone”. If the participant responded 1 “alone” in all the above questions, then he/she was coded into the category 1 “mobile”; otherwise, 0 “not mobile”.

Decision making power (Cronbach alpha: 0.94): The variable was assessed using the following questions: Who in your family usually has the final say on A) Making large household purchases? B) Making household purchases for daily needs? C) Decisions about major family investments? D) Visits to family and/or relatives/friends? E) What food should be cooked each day? F) Your families healthcare? G) Your own healthcare? H) Choices around contraceptives/family planning? The response for the above questions were coded as 1 “Mainly respondent”, 2 “Mainly another women family member”, 3 “Mainly another men family member” and 4 “Respondent and family member(s) jointly”. The variables were coded as 1 if “mainly respondent” and else 0 “mainly another women family member/ mainly another men family member/ respondent and family member(s) jointly”. If the participant responded 1 “mainly respondent” in all the above questions, then he/she was coded in category 1 “have decision making power”; otherwise, 0 “have no decision-making power”.

Economic resources (Cronbach alpha: 0.73): The variable was assessed using the following questions: A) Do you, in your own name, own any land, your homestead land, or your house? B) Do you yourself own any productive assets (for example, cattle, sewing machine, etc.)? C) Do you have any cash as savings? D) Have you ever used your savings for business or money-lending? The responses of the above question were coded as 0 “no” and 1 “yes”. If the respondent responded 1 “yes” in all the above questions, then he/she was coded into the category 1 “have economic resources”; otherwise, 0 “have no economic resources”.

### Other explanatory variables

#### Respondent’s characteristics

Age in years, educational status of the respondent (not educated/ primary completed, secondary completed, senior secondary completed, graduate and above), working status of the respondent (yes and no), alcohol consumption by the respondent (yes and no), ever had children (yes and no).

### Spousal characteristics

Spousal controlling behavior was coded using the following questions: A) Tries to keep you away from seeing your friends? B) Tries to restrict contact with your family of birth? C) Insists on knowing where you are at all times? D) Ignores you and treats you indifferently? E) gets angry if you speak with another man/woman? F) is often suspicious that you are unfaithful? G) expects you to ask his/her permission before seeking health care for yourself? H) Prevents you from expressing your opinion in public? The responses of the above question were coded as 0 “no” and 1 “yes”. If the particioant responded 1 “yes” in any of the questions, then he/she was coded into the category 1 “controlling behavior”; otherwise, 0 “no controlling behavior”. Other factors were spousal age gap in years, educational status of the spouse (not educated/primary completed, secondary completed, senior secondary completed, graduate and above), working status of the spouse (yes and no) and alcohol consumption by spouse (yes and no).

### Household characteristics

Religion (Hindu and others), caste (non-Scheduled Caste/Scheduled Tribe (SC/ST) or (SC/ST), income in Indian national rupees (INR), below poverty line status (yes or no), family structure (nuclear or joint/extended) and districts (Jaipur or Baharaich).

### Sampling strategy and sample size

A line listing exercise was carried out to prepare the sampling frame for selecting the quantitative sample. Using the sample frame, the required number of respondents were selected from the study with stratified random sampling. The sample size was measured by using the proportion of IPV in Uttar Pradesh as 36.7% from NFHS 4 (as NFHS 5 data had not come when the sample was being calculated) (p equals 0.384), z-value of 1.96 and level of precision as 5%. The calculated sample size came out to be 363 cases. After adjusting for 10% of non-response rate, the effective sample size was estimated to be 400 cases. The total sample for the two districts, Baharaich and Jaipur, was 800. However, the response rate was 72.3%, and therefore, the total sample for the analysis was 578 married youth aged 15–24 years. The COVID-19 pandemic affected reaching the desired sample size. Inclusion criteria of the study was youth in the age group of 15–24 years and their parents. All individuals who did not provide consent to participate in the study were excluded. Moreover, all those individuals who had suffered a major illness in the past 1 year were also excluded.

### Data collection and management

The data was collected through a semi-structured questionnaire. The questionnaire comprised of variables on socio-demographic factors, knowledge and practices on gender equitable norms, beliefs about gender, decision making, economic security and contribution, GBV (physical, sexual) and controlling behaviour of the spouse. The questionnaire was translated to the local language, Hindi, and pre-tested in the field during the training session towards finalization. The data collection was conducted by a team of 12 investigators comprising of both men and women, to consider sensitivity of the issues addressed. The investigators were trained for a 3-day period through an online platform. They were trained on various techniques to conduct the survey, and emphasis was given to achieve the maximum intra and inter-individual agreement with respect to all the measurements. The data collection team, including field managers and surveyors, were also trained on ethical protection, consent/assent process, working with youth, and to adhere to the ethical guidelines.

During the training, the teams also carried out mock surveys. Detailed information with the aim, objectives, risks, benefits with implications of the study were explained to the youth before conducting the study. Informed consent was obtained from all subjects and/ or their legal guardian(s). Written informed consent was taken from the parents for confirmation of their enrolment in the study. For participants above 18 years, consent forms were distributed to explain the purpose of the study to ensure full understanding of the participants and provide opportunity to opt-out from the study if they so wished. The participants were assured that their participation in the study is completely voluntary and they could choose to withdraw from it at any point, if they feel uncomfortable in answering any question.

The quantitative data was collected through surveyors via mobiles/tablets. The data was collected from the youth (men and women) from the community who were not couples. Additionally, to avoid risks associated with the disclosure of sensitive information, password-protected files were maintained, and all the data records were kept confidential. Personal identifiers were kept separate from the main data collection tool and unique ID codes for each participant were generated. Completed questionnaires were kept locked in a dedicated storage facility and access was limited to the principal investigator and to the data management personnel only. Ethical approval was obtained from the institutional review board of MAMTA Health Institute for Mother and Child, the main coordinating organization of project SAWERA.

### Statistical analysis

Descriptive statistics [mean (standard deviation), percentage], along with bivariate analysis (percentage), were conducted through STATA 14 [20] to present the results. The Chi-squared test [21] and proportion test [22] were used to test the level of significance during bivariate analysis and gender differences respectively. Additionally, binary logistic regression analysis [23] was used to present the estimates in the form of Adjusted Odds Ratio (aOR) at a 95% confidence interval. The analysis was segregated by sex of the respondent. Variance inflation factor [24] was estimated to check multi-collinearity between the variables, and it was found that there was no evidence of multi-collinearity between the variables.

## Results

Table 1 presents the socio-economic profile of the respondents of the study. The mean age of the respondents was 22.7 years for men and 21.8 years for women. About one-fifth of the men and women were not educated and/or their primary schooling was not completed. About 76.1% and 12.4% of the men and women respectively were currently working. Almost 5.5% and 1.2% of the men and women respectively had ever consumed alcohol. The mean spousal age gap was 1.3 years for men and 2.9 years for women. About 56% of the spouses were not educated and/or their primary schooling was not completed. About 33.3% and 63.2% of the men and women respectively reported that their spouses were currently working. Only, 1.9% and 11.8% of the men and women respondents respectively reported that their spouses consumed alcohol.

**Table 1:** Socio-economic characteristics of the study population, (*n* = 578)

Background characteristics	Men		Women	
	***N***	**%**	***N***	**%**
**Respondent characteristics**				
**Age of the respondent in years [mean (sd)]**	22.7 (1.6)		21.8 (2.0)	
**Educational status of respondent**				
Not educated/Primary completed	54	21.2	73	22.6
Secondary completed	75	29.4	76	23.5
Senior secondary completed	36	14.1	30	9.3
Graduate and above	90	35.3	144	44.6
**Working status of the respondent**				
Yes	194	76.1	40	12.4
No	61	23.9	283	87.6
**Alcohol consumption of respondent**				
Yes	14	5.5	3	1.2
No	241	94.5	252	98.8
**Ever had children**				
Yes	144	56.5	190	58.8
No	111	43.5	133	41.2
**Spousal characteristics**				
**Spousal age gap in years [mean (sd)**	-1.3 (1.7)		2.9 (2.8)	
**Spouse controlling behaviour**				
No	209	82.0	190	58.8
Yes	46	18.0	133	41.2
**Educational status of spouse**				
Not educated/Primary completed	143	56.1	181	56.0
Secondary completed	72	28.2	78	24.2
Senior secondary completed	29	11.4	29	9.0
Graduate and above	11	4.3	35	10.8
**Working status of spouse**				
Yes	85	33.3	204	63.2
No	170	66.7	119	36.8
**Alcohol consumption of spouse**				
Yes	6	1.9	38	11.8
No	317	98.1	285	88.2
**Household characteristics**				
**Religion**				
Hindu	173.0	67.8	228.0	70.6
Others	82.0	32.2	95.0	29.4
**Caste**				
Non-SC/ST	177	69.4	223	69.0
SC/ST	78	30.6	100	31.0
**Income in INR [mean (sd)**	6518 (4078)		8778 (10,011)	
**Below poverty line status**				
No	185	72.6	250	77.4
Yes	70	27.5	73	22.6
**Family structure**				
Nuclear	115	45.1	131	40.6
Joint or extended	140	54.9	192	59.4
**Districts**				
Baharaich	181	71.0	196	60.7
Jaipur	74	29.0	127	39.3

*INR *Indian national rupee*, sd *Standard deviation*, n *Sample*, % *Percentage

**Fig. 2:**
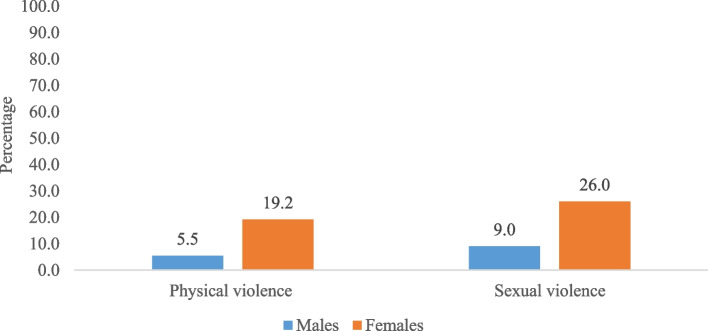
Percentage of men and women who reported physical and sexual violence

Figure 2 represents the percentage of men and women who reported to have experienced physical and sexual violence. It was found that 5.5% and 19.2% (difference: 13.7%; *p* < 0.001), 9.0% and 26.0% (difference: 17%; *p* < 0.001), of men and women respectively had experienced physical and sexual violence.

### Proportion test was used to check the significance level for men and women estimates

**Fig. 3:**
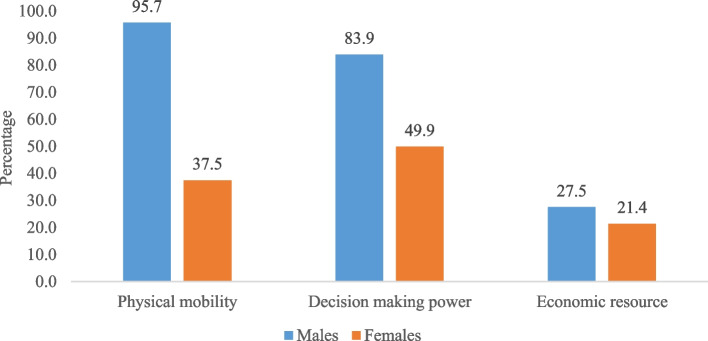
Percentage of men and women who had physical mobility, decision making power and economic resources

Figure 3 represents the percentage of men and women who had physical mobility, decision making power and economic resources. It was revealed that 95.7% and 37.5% (difference: 58.2%; *p* < 0.001), 83.9% and 49.9% (difference: 34%; *p* < 0.001), 27.5% and 21.4% (difference: 6.1%; *p* < 0.089) of men and women respectively had physical mobility, decision making power and economic resources.

### Proportion test was used to check the significance level for men and women estimates

Table 2 represents the association of empowerment indicators with physical and sexual violence among young men and women. It was found that higher percentage of men with no mobility had experienced physical violence (18.2% vs 4.9%; *p*-value: 0.059). Higher percentage of men with economic resources experienced physical violence (10.0% vs 3.8%; *p*-value: 0.071) whereas in case of women the results were the opposite. Higher percentage of women who were mobile experienced sexual violence (33.9% vs 21.3%; *p*-value: 0.012). Higher percentage of men (15.7% vs 6.5%; *p*-value: 0.022) and women (52.2% vs 18.9%; *p*-value: < 0.001) who had economic resources experienced sexual violence.

**Table 2:** Association of empowerment indicators with physical and sexual violence among young men and women, (*n* = 578)

Variables	Physical violence				Sexual violence			
	**Men**		**Women**		**Men**		**Women**	
	**%**	***p*-value**	**%**	***p*-value**	**%**	***p*-value**	**%**	***p*-value**
**Empowerment indicators**								
** Physical mobility**		0.059		0.217		0.278		0.012
No	**18.2**		21.3		18.2		**21.3**	
Yes	**4.9**		15.7		8.6		**33.9**	
**Decision making power**		0.851		0.412		0.312		0.774
No	4.9		21		4.9		25.3	
Yes	5.6		17.4		9.8		26.7	
**Economic resource**		0.052		0.071		0.022		< 0.001
No	**3.8**		**21.3**		**6.5**		**18.9**	
Yes	**10.0**		**11.6**		**15.7**		**52.2**	
**Respondent characteristics**								
**Age of the respondent in years**	0.05	0.382	0.1	0.271	-0.04	0.502	-0.07	0.189
**Educational status of respondent**		0.181		0.015		0.456		0.405
Not educated/Primary completed	1.9		20.6		3.7		31.5	
Secondary completed	9.3		9.2		10.7		23.7	
Senior secondary completed	8.3		10.0		8.3		33.3	
Graduate and above	3.3		25.7		11.1		22.9	
**Working status of the respondent**		0.385		0.771		0.799		0.031
Yes	6.2		17.5		8.8		**40.0**	
No	3.3		19.4		9.8		**24.0**	
**Alcohol consumption by respondent**		0.007		0.375		0.009		0.176
Yes	**21.4**		33.3		**28.6**		50.0	
No	**4.6**		18.9		**7.9**		25.6	
**Ever had children**		0.291		0.879		0.188		0.163
Yes	4.2		19.5		6.9		23.2	
No	7.2		18.8		11.7		30.1	
**Spousal characteristics**								
**Spousal age gap in years**	-0.003	0.958	0.002	0.607	0.070	0.291	-0.001	0.993
**Spouse controlling behavior**		< 0.001		< 0.001		< 0.001		< 0.001
No	**2.9**		**10.0**		**2.9**		**17.4**	
Yes	**17.4**		**32.3**		**37.0**		**38.4**	
**Educational status of spouse**		0.064		0.005		0.045		0.587
Not educated/Primary completed	**2.1**		**26.0**		**5.6**		28.2	
Secondary completed	**9.7**		**11.5**		**9.7**		25.6	
Senior secondary completed	**10.3**		**13.8**		**20.7**		24.1	
Graduate and above	**9.1**		**5.7**		**18.2**		17.1	
**Working status of Spouse**		0.052		0.003		0.757		0.589
Yes	**3.5**		**27.7**		9.4		27.7	
No	**9.4**		**14.2**		8.2		25.0	
**Alcohol consumption by Spouse**		0.033		0.012		0.139		0.220
Yes	**33.3**		**34.2**		33.3		34.2	
No	**5.2**		**17.2**		8.7		24.9	
**Household characteristics**								
**Religion**		0.039		< 0.001		0.012		0.935
Hinduism	**7.5**		**12.7**		**12.1**		25.9	
Others	**1.2**		**34.7**		**2.4**		26.3	
**Caste**		0.001		0.028		< 0.001		0.999
Non-SC/ST	**2.3**		**22.4**		**4.5**		26.0	
SC/ST	**12.8**		**12.0**		**19.2**		26.0	
**Income (INR)**	**0.30**	< 0.001	0.02	0.321	**0.20**	< 0.001	0.10	0.220
**Below poverty line status**		0.603		0.018		0.257		0.541
No	6.0		**16.4**		10.3		25.2	
Yes	4.3		**28.8**		5.7		28.8	
**Family structure**		0.042		< 0.001		0.248		0.449
Nuclear	**8.7**		**29.0**		11.3		28.2	
Joint or extended	**2.9**		**12.5**		7.1		24.5	
**Districts**		< 0.001		< 0.001		< 0.001		0.302
Bahraich	**1.1**		**27.6**		**4.4**		24.0	
Jaipur	**16.2**		**6.3**		**20.3**		29.1	

*p*-value based on chi-square test

Table 3 represents the logistic regression estimates for empowerment indicators with physical and sexual violence among young men and women. Men with no mobility [aOR: 4.00; CI: 00.28,58.01], no decision-making power [aOR: 4.96; CI: 0.32,76.37] and no economic resources [aOR: 2.31; CI: 0.36,14.81] showed a higher likelihood to experience physical violence in but was not statistically significant. Women with no decision-making power [aOR: 2.12; CI: 1.01,4.43] had a higher likelihood to experience physical violence in comparison to their counterparts. Women with no mobility [aOR: 0.49; CI: 0.26, 0.92] and no economic resources [aOR: 0. 19; CI: 0.09,0.39] had a significantly lower likelihood to experience sexual violence in comparison to men. However, women with no decision-making power [aOR: 1.96; CI: 1.02,3.77] had significantly higher odds for sexual violence in reference to women with decision making power.

**Table 3:** Logistic regression estimates for empowerment indicators with physical and sexual violence among young men and women, (n = 578)

Variables	Physical violence AOR (95% CI)		Sexual violence AOR (95% CI)	
	**Men**	**Women**	**Men**	**Women**
**Empowerment indicators**				
** Mobility**				
No	4.00(0.28,58.01)	1.33(0.61,2.89)	0.94(0.08,10.94)	0.49*(0.26,0.92)
Yes	Ref	Ref	Ref	**Ref**
**Decision making power**				
No	4.96(0.32,76.37)	2.12*(1.01,4.43)	0.74(0.11,5.2)	1.96*(1.02,3.77)
Yes	Ref	**Ref**	Ref	**Ref**
**Economic resource**				
No	2.31(0.36,14.81)	1.80(0.66,4.9)	1.15(0.25,5.35)	0.19*(0.09,0.39)
Yes	Ref	Ref	Ref	**Ref**
**Respondent characteristics**				
**Age of the respondent in years**	1.24(0.61,2.52)	1.08(0.9,1.3)	1.03(0.69,1.55)	0.99(0.85,1.16)
**Educational status of respondent**				
Not educated/Primary completed	Ref	Ref	Ref	Ref
Secondary completed	1.53(0.02,121.44)	1.04(0.3,3.69)	0.57(0.04,7.4)	0.43(0.17,1.1)
Senior secondary completed	1.05(0.01,108.85)	0.58(0.11,3.19)	0.21(0.01,4.96)	0.85(0.25,2.89)
Graduate and above	1.60(0.02,114.6)	1.14(0.5,2.57)	1.52(0.17,13.9)	0.52(0.24,1.12)
**Working status of the respondent**				
Yes	Ref	Ref	Ref	Ref
No	2.7(0.24,30.05)	0.87(0.27,2.75)	4.26(0.66,27.7)	0.93(0.39,2.22)
**Alcohol consumption by respondent**				
Yes	Ref	Ref	Ref	Ref
No	0.56(0.04,7.19)	2(0.18,21.82)	0.25(0.03,1.92)	0.52(0.06,4.56)
**Ever had children**				
Yes	Ref	Ref	Ref	**Ref**
No	2.22(0.5,9.82)	1.01(0.48,2.11)	1.94(0.53,7.11)	2.09*(1.06,4.1)
**Spousal characteristics**				
**Spousal age gap in years**	1.14(0.73,1.79)	1.01(0.89,1.15)	1.44*(1.05,1.98)	1.01(0.9,1.12)
**Spouse controlling behaviour**				
No	Ref	**Ref**	**Ref**	**Ref**
Yes	5.02(0.67,37.82)	3.79*(1.75,8.19)	47.24*(8.12,274.92)	4.03*(2.09,7.79)
**Educational status of spouse**				
Not educated/Primary completed	Ref	Ref	Ref	Ref
Secondary completed	1.93(0.24,15.46)	0.84(0.31,2.29)	0.75(0.12,4.76)	0.73(0.32,1.63)
Senior secondary completed	0.92(0.06,14.15)	1.44(0.32,6.43)	2.23(0.32,15.53)	0.9(0.27,3.04)
Graduate and above	1.82(0.09,35.84)	0.52(0.1,2.67)	0.58(0.04,9.24)	0.32(0.09,1.12)
**Working status of Spouse**				
Yes	Ref	Ref	Ref	Ref
No	3.57(0.49,26.04)	0.65(0.32,1.31)	0.68(0.13,3.61)	0.79(0.41,1.52)
**Alcohol consumption by Spouse**				
Yes	Ref	Ref	Ref	Ref
No	0.01*(0,0.52)	0.20*(0.06,0.6)	1.31(0.04,42.56)	1.14(0.42,3.06)
**Household characteristics**				
**Religion**				
Hinduism	Ref	**Ref**	Ref	Ref
Others	0.33(0.02,5.69)	2.32*(1.07,5.03)	0.92(0.13,6.8)	0.97(0.46,2.03)
**Caste**				
Non-SC/ST	Ref	Ref	Ref	Ref
SC/ST	1.55(0.23,10.55)	1.27(0.51,3.12)	3.3(0.61,17.86)	1.02(0.5,2.1)
**Income (INR)**	1.01(0.97,1.06)	1.03(0.99,1.005)	1.00(0.90,1.09)	1.04(0.89,1.12)
**Below poverty line status**				
No	Ref	Ref	Ref	Ref
Yes	14.08(0.85,234.24)	1.51(0.69,3.30)	1.42(0.24,8.45)	1.43(0.67,3.06)
**Family structure**				
Nuclear	Ref	Ref	Ref	Ref
Joint or extended	0.68(0.04,11.53)	0.52(0.26,1.07)	5.46(0.68,43.59)	1.03(0.54,1.95)
**Districts**				
Baharaich	**Ref**	**Ref**	Ref	Ref
Jaipur	62.18*(1.48,2610.33)	0.31*(0.1,0.99)	7.50(0.75,74.64)	1.85(0.76,4.51)

*Ref *Reference

*if *p *0.05, the analysis was adjusted for respondents’, spousal and household characteristics

Through review of literature and according to the existing variables we took some individual, socio economic and spousal characteristics that could lead to GBV such as alcohol consumption, spousal controlling behavior, below poverty line status, ever had children, spousal age gap, religion etc. We found women who did not have a child were [aOR: 2.09; CI: 1.06,4.1] more likely to go through sexual violence. If there were spousal controlling behavior women were three times [aOR: 3.79; CI: 1.75,8.19] more likely to go through physical violence. On the other hand women were four times more likely [aOR: 4.03; CI: 2.09,7.79] to go through sexual violence. We also found that women who came from other ethnic backgrounds had higher odds [aOR: 2.32; CI: 1.07,5.03] of going through physical violence.

We did additional analysis (Supplementary Table 1) for all the variables to see the differences in GBV in the rural (Bahraich) and urban settings (Jaipur). In the rural setting, we found that women had significantly higher likelihood to go through physical violence [aOR: 9.15; CI: 1.22,68.48]. The results showed if there is spousal controlling behavior the participants were two times more likely to go through physical violence [aOR: 2.76; CI: 1.23,6.18] and five times more likely to go through sexual violence [aOR: 5.73; CI: 2.55,12.87]. Coming from SC/ST had a higher likelihood to go through sexual violence in a rural setting [aOR: 3.07; CI: 1.27,7.43].

In the urban setting of Jaipur (Supplementary Table 2) it was found that if the respondents had no economic resources there was a higher likelihood of physical violence [aOR: 5.36; CI: 1.13,25.2]. Women were six times more likely to go through sexual violence [aOR: 6.11; CI: 1.10,33.84]. If there were spousal controlling behavior participants were thirteen times more likely to go through physical violence [aOR: 13.37; CI: 3.05,58.54] and six times more likely to go through sexual violence [aOR: 6.32; CI: 2.50,15.96].

## Discussion

This study examined the relationship between WEE indicators, such as physical mobility, decision making and economic empowerment, and their association with GBV among married youth (15–24 years) living in urban and rural parts of Uttar Pradesh and Rajasthan. The study highlights the centrality of marriage as a setting where various influences intersect, particularly gender inequality and lack of WEE, which lays a foundation to women’s vulnerability. It was also explored how the socio-economic factors, namely spousal characteristics and ethnic characteristics are associated with GBV.

In our study we found that nearly 19.2% of women experienced physical violence compared to the 5.5% of men. Additionally, 26% of the women experienced sexual violence compared to the 9% of men. Considering, the progress being made in India with increasing education and overall development over the years, such stark gender differentials is still a concern.

In India, due to the deep-rooted gender inequalities in the society, controlling behavior by the husband is evident from the high prevalence of IPV and other GBV indicators in the national data [11]. In this study, we found that women who experienced controlling behavior by their spouse were three times at a higher risk of physical violence and four times at a higher odds of sexual violence. This finding corroborates similar findings from other countries such as Spain, Bangladesh and Nigeria [25–27]. In India, due to the patriarchal construct of marriages, men show their dominance and non-egalitarian gender norms where women need to be subservient and act according to the husband’s wishes. More so, when a woman is economically dependent on her spouse, is less educated, her spouse restricts her interaction with other people and resorts to violence if his needs are not met.

In our study, we found that women not having physical mobility has a negative relationship with sexual violence or women with physical mobility had a higher odds of going through sexual violence. This is due to the biased gender and social norms prevalent in the society, where violence is happening not just by the husband but also by other perpetrators like relatives or acquaintances. There is prior evidence from studies that investigated social norms regarding sexual violence, where most often girls are blamed if violence occurs for reasons like inappropriate clothing or inciting behavior [28]. Further, it is considered normal that women restrict their movement and actions to prevent men from assaulting them [29].

Further, we found that women who had more economic resources were at a higher odds of sexual violence. This finding is similar to a previous study conducted in Nigeria which showed that women who are earning are more prone to physical violence and sexual violence [15]. Moreover, women who do not own economic resources nor are able to negotiate change, are dependent on their husbands. This makes them more vulnerable to controlling behavior by their husbands. In India, boys get socialized in a patriarchal context, where they associate control over their wives as a masculine trait. Additionally if these men themselves are undergoing financial stress, it may threaten their own abilities and this can lead them to become more controlling and violent towards their partners [30].

We also found that women who did not have children were at a higher risk of sexual violence. A possible reason for this could be that early marriage makes them more vulnerable to sexual violence [31] and having children might be considered as a protective factor against sexual violence, committed by an outside perpetrator. Another, speculative reason for this association could be that a small proportion of this sample are women who themselves cannot have a child or their husbands cannot due to medical reasons, such as infertility. There is evidence from a study in India which showed 7.8% prevalence of spousal physical/sexual violence among childless women, compared to 6.1% among women who have children [32].

Socio-economic factors showed an effect on violence in our study. Women in rural areas were more likely to be exposed to physical violence, which is in line with previous studies conducted in India in Puducherry (56.7%) and Haryana (37%), which showed that married women have experienced more domestic violence [33, 34]. Our additional analysis shown in the supplementary table shows that spousal controlling behavior was significantly associated with physical and sexual violence in urban and rural areas. Additionally, coming from a more backward section SC/ST had a higher association with sexual violence. Moreover, previous research shows that lower socio-economic status is a determinant of physical violence. This could be due to accompanying factors such as alcohol use, job dissatisfaction, frustration of limited resources and patriarchal upbringing, among others [35]. Even though in the present study factors such as below the poverty line status and lower income did have an association in the bivariate analysis, but no association was observed in the multi-variate model due to confounding factors.

Alcohol consumption by the spouse was a factor that was significantly associated with physical violence in men and women. Evidence from various studies conducted in India shows that alcohol consumption along with factors such as having multiple sexual partners, poorer backgrounds, lower caste, and women having greater economic autonomy are associated with GBV [36–38].Most of these studies have shown an effect on VAW, but in our study, we have shown data on violence in men too. Religious and ethnic differences were also found. Muslim families were at a higher risk of physical violence. This finding is in line with evidence from a previous study conducted in Mumbai slums, which showed that women who were from lower economic backgrounds, were from Muslim families and whose husbands consumed alcohol were at a higher risk of GBV [39].

In light to gravity of the situation, GTA was considered as a suitable approach for SAWERA project. There is mounting global evidence which suggests programs on men engagement incorporating a “gender transformative” approach are more likely to shift men’s gender and violence-related attitudes and behaviors than programs that do not explicitly address gender norms [40]. A recent review of literature on GTA in the context of GBV showed that 6 out of 10 studies showed statistically significant impact on at least one of the following outcomes: increase in gender equitable attitudes and care or domestic work and decreases in reported IPV and social acceptance of IPV [41]. This approach is being used widely, as another, systematic review published in the lancet, adopted GTA among children, adolescents and adults, with a focus on sexual and reproductive health, HIV, and violence which showed that many of these approaches are effective in them [42]. Thus, we used this approach to reduce violence and improve its related outcomes.

### Strength and limitations

One of the assets of the study is that it investigated married youth – a neglected population – and looked into violence against both men and women (important to note that not many studies give data on violence against men). Additionally, we specifically investigated positive and progressive factors of women’s empowerment which are associated with GBV to generate scientific evidence for action. One limitation of the study was its cross-sectional nature, due to which it is challenging to explain the causality and temporal ordering of the determinants with the study outcomes, as it is well established that factors like economic empowerment of women can be a cause as well as a consequence of IPV [43]. Secondly, self-reported data on sexual violence may be subject to recall bias. While this study focused on physical and sexual violence, future studies should focus on emotional and economic violence. We used the term GBV more generally than IPV as it would have led to under reporting due to the issue of giving socially desirables responses. As it comprised of sensitive issues like physical and sexual violence which are usually under reported, Thirdly, generalizability of the study can be another limitation as the data is collected only from two districts in two states of India. However, societal issues in unequal gender norms and violence in India are not too different across the country, in the rural and urban settings which is evident from this study. Fourthly, one of the variables on physical mobility does not reflect the actual agency or empowerment to go to a particular place but rather reflects more on the experience of doing it. The questionnaire was based on various literature and on the NFHS data set, hence that might be a limiting factor. The final limitation of this study was that even though disability aggregated data was collected, the numbers of that sample were too low to analyze it further.

## Conclusion

The study highlights the complex interplay of women’s empowerment indicators with GBV and how socio-demographic and economic factors interplay to confound this relationship. It was evident from the results that to empower women, it is crucial to include men – a largely overlooked population – and make them the focus of studies and programs. It is vital to understand the factors that lead to physical and sexual violence by using methods such as GTA. There is a need to work towards more proximate factors that may influence GBV, including alcohol consumption, ethnic and rural background, economic empowerment of women, agency, physical mobility and decision-making. This study has highlighted the dire need to work on biased gender norms and critically reflect on the negative consequences of gender inequality, and to think of alternate ways of how we see masculine and feminine roles in our society.

It is crucial to work towards improving women’s status and autonomy at the individual and community level by working with all genders and key stakeholders to address VAW. For further research, we recognize the need for more efforts to bring about equitable gender norms, which would lay a foundation for improved health and well-being over the life course starting from early adolescence [42]. So far, such programs that focus on scale and impact have been limited.

## Supplementary Information

**Additional file 1: Supplementary tables.**

## Data Availability

The datasets used and/or analyzed during the current study are available from the corresponding author on reasonable request.

## Abbreviations

AOR: Adjusted odds ratio
GBV: Gender based violence
GTA: Gender transformative approach
HIV: Human immunodeficiency viruses
IPV: Intimate partner Violence
NFHS: National Family Health Survey
OR: Odds ratio
SC: Scheduled caste
SDG: Sustainable development goals
STI: Sexually transmitted infections
ST: Scheduled tribe
VAW: Violence against women
WEE: Women’s economic empowerment
WHO: World Health Organization
