# Supplementary material for: Effect of physical mobility, decision making and economic empowerment on gender-based violence among married youth in India-SAWERA project
Source: BMC Public Health. 2023 Mar 23;23:548. doi: 10.1186/s12889-023-15421-4 (PMC10034242; doi:10.1186/s12889-023-15421-4)
Supplement: Supplementary file 1 — Additional file 1: Supplementary tables. [file 12889_2023_15421_MOESM1_ESM.docx]

**Supplementary Table 1**

| Logistic regression estimates for empowerment indicators with physical and sexual violence among rural population (Bahraich), (n=377) | | |
| --- | --- | --- |
| **Variables** | **Physical violence**  **AOR (95% CI)** | **Sexual violence**  **AOR (95% CI)** |
|  | **All** | **All** |
| **Empowerment indicators** |  |  |
| **Mobility** |  |  |
| No | 1.66(0.67,4.11) | 1.05(0.42,2.61) |
| Yes | Ref. | Ref. |
| **Decision making power** |  |  |
| No | 1.59(0.73,3.46) | 1.36(0.61,3.06) |
| Yes | Ref. | Ref. |
| **Economic resource** |  |  |
| No | 1.43(0.46,4.41) | 0.55(0.20,1.49) |
| Yes | Ref. | Ref. |
| **Respondent characteristics** |  |  |
| **Age of the respondent in years** | 1.06(0.87,1.28) | 0.88(0.74,1.06) |
| **Educational status of respondent** |  |  |
| Not educated/Primary completed | Ref. | Ref. |
| Secondary completed | 1.29(0.29,5.70) | 0.31(0.06,1.50) |
| Senior secondary completed | 0.65(0.10,3.96) | 0.13(0.01,1.44) |
| Graduate and above | 1.19(0.51,2.75) | 0.99(0.44,2.20) |
| **Working status of the respondent** |  |  |
| Yes | Ref. | Ref. |
| No | 1.51(0.39,5.78) | 1.26(0.42,3.77) |
| **Sex** |  |  |
| Male | Ref | Ref |
| Female | **9.15 (1.22,68.48)** | 2.33 (0.51,10.57) |
| **Alcohol Consumption by the respondent** |  |  |
| Yes | Ref. | Ref. |
| No | 0.81(0.08,7.95) | 0.65(0.09,4.48) |
| **Ever had children** |  |  |
| Yes | Ref. | Ref. |
| No | 0.86(0.40,1.85) | 1.82(0.84,3.93) |
| **Spousal characteristics** |  |  |
| **Spousal age gap in years** | 1.02(0.89,1.18) | 1.06*(0.92,1.21) |
| **Spouse controlling behaviour** |  |  |
| No | Ref. | **Ref.** |
| Yes | **2.76(1.23,6.18)** | **5.73*(2.55,12.87)** |
| **Educational status of spouse** |  |  |
| Not educated/Primary completed | Ref. | Ref. |
| Secondary completed | 0.78(0.25,2.43) | 0.94(0.29,3.04) |
| Senior secondary completed | 1.94(0.39,9.57) | 1.58(0.33,7.42) |
| Graduate and above | 0.66(0.12,3.54) | 1.19(0.27,5.20) |
| **Working status of Spouse** |  |  |
| Yes | Ref. | Ref. |
| No | 0.84(0.40,1.79) | 0.63(0.29, 1.38) |
| **Alcohol consumption by Spouse** |  |  |
| Yes | Ref. | Ref. |
| No | 0.20 (0.05,0.80) | 0.47(0.12,1.86) |
| **Household characteristics** |  |  |
| **Religion** |  |  |
| Hinduism | Ref. | Ref. |
| Others | 1.74(0.80,3.79) | 0.60(0.26,1.36) |
| **Caste** |  |  |
| Non-SC/ST | Ref. | Ref. |
| SC/ST | 1.36(0.50,3.69) | **3.07(1.27,7.43)** |
|  |  |  |
| **Income (INR)** | 1.00(0.99,1.00) | 1.00(0.99,1.00) |
| **Below poverty line status** |  |  |
| No | Ref. | Ref. |
| Yes | 1.51(0.71,3.21) | 2.08(0.98,4.43) |
| **Family structure** |  |  |
| Nuclear | Ref. | Ref. |
| Joint or extended | 0.54(0.26,1.13) | 0.94(0,43, 2.01) |
|  |  |  |
| *Ref: Reference; *if p<0.05; the analysis was adjusted for respondents’, spousal and household characteristics* | | |

**Supplementary Table 2**

| Logistic regression estimates for empowerment indicators with physical and sexual violence among urban population (Jaipur), (n=201) | | |
| --- | --- | --- |
| **Variables** | **Physical violence**  **AOR (95% CI)** | **Sexual violence**  **AOR (95% CI)** |
|  | **All** | **All** |
| **Empowerment indicators** |  |  |
| **Mobility** |  |  |
| No | 1.04(0.17,6.22) | 0.25(0.09,0.72) |
| Yes | Ref. | Ref. |
| **Decision making power** |  |  |
| No | 5.33(0.94,30.0) | 2.42(0.90,6.50) |
| Yes | Ref. | Ref. |
| **Economic resource** |  |  |
| No | **5.36(1.13,25.2)** | 1.15(0.05,0.42) |
| Yes | Ref. | Ref. |
| **Respondent characteristics** |  |  |
| **Age of the respondent in years** | 1.19(0.74,1.89) | 1.25(0.95,1.63) |
| **Educational status of respondent** |  |  |
| Not educated/Primary completed | Ref. | Ref. |
| Secondary completed | 1.22(0.12,12.0) | 0.43(0.12,1.56) |
| Senior secondary completed | 0.68(0.05,9.14) | 0.14(0.23,5.59) |
| Graduate and above | 0.73(0.07,7.44) | 0.37(0.08,1.57) |
| **Working status of the respondent** |  |  |
| Yes | Ref. | Ref. |
| No | 0.49(0.08,2.98) | 1.32(0.43,4.08) |
| **Sex** |  |  |
| Male | Ref | Ref |
| Female | .077(.003, 1.64) | **6.11 (1.10,33.84)** |
| **Alcohol consumption by respondent** |  |  |
| Yes | Ref. | Ref. |
| No | 0.11(0.01,1.32) | 0.17(0.01,2.01) |
| **Ever had children** |  |  |
| Yes | Ref. | Ref. |
| No | 3.22(0.89,11.63) | 3.35(1.28,8.78) |
| **Spousal characteristics** |  |  |
| **Spousal age gap in years** | 1.19(0.91,1.57) | 0.99*(0.82,1.19) |
| **Spouse controlling behaviour** |  |  |
| No | Ref. | **Ref.** |
| Yes | **13.37(3.05,58.54)** | **6.32*(2.50,15.96)** |
| **Educational status of spouse** |  |  |
| Not educated/Primary completed | Ref. | Ref. |
| Secondary completed | 2.06(0.34,12.27) | 0.90(0.30,2.67) |
| Senior secondary completed | 1.75(0.17,17.17) | 1.17(0.26,5.24) |
| Graduate and above | 1.00(0.06,14.93) | 0.26(0.05,1.32) |
| **Working status of Spouse** |  |  |
| Yes | Ref. | Ref. |
| No | 0.70(0.16,3.01) | 0.73(0.28,1.91) |
| **Alcohol consumption by Spouse** |  |  |
| Yes | Ref. | Ref. |
| No | 0.95 (0.01,0.89) | 3.07(0.68,13.70) |
| **Household characteristics** |  |  |
| **Religion** |  |  |
| Hinduism | Ref. | Ref. |
| Others | 6.85(0.75,6.20) | 1.49(0.32,6.84) |
| **Caste** |  |  |
| Non-SC/ST | Ref. | Ref. |
| SC/ST | 3.22(0.63,16.45) | 0.96(0.37,2.49) |
|  |  |  |
| **Income (INR)** | 1.00(0.99,1.00) | 1.00(0.99,1.00) |
| **Below poverty line status** |  |  |
| No | Ref. | Ref. |
| Yes | 3.48(0.35,33.80) | 1.13(0.00,2.60) |
| **Family structure** |  |  |
| Nuclear | Ref. | Ref. |
| Joint or extended | 0.67(0.11,4.14) | 2.24(0.80,6.25) |
|  |  |  |
| *Ref: Reference; *if p<0.05; the analysis was adjusted for respondents’, spousal and household characteristics* | | |
